# Towards an effective control programme of soil-transmitted helminth infections among Orang Asli in rural Malaysia. Part 1: Prevalence and associated key factors

**DOI:** 10.1186/1756-3305-6-27

**Published:** 2013-01-28

**Authors:** Nabil A Nasr, Hesham M Al-Mekhlafi, Abdulhamid Ahmed, Muhammad Aidil Roslan, Awang Bulgiba

**Affiliations:** 1 Department of Parasitology, Faculty of Medicine, University of Malaya, 50603, Kuala Lumpur, Malaysia; 2 Department of Biology, Faculty of Natural and Applied Sciences, Umaru Musa Yar’adua University, Katsina, Katsina State, Nigeria; 3 Julius Centre University of Malaya, Department of Social & Preventive Medicine, Faculty of Medicine, University of Malaya, 50603, Kuala Lumpur, Malaysia; 4 Department of Parasitology, Faculty of Medicine and Health Sciences, Sana’a University, Sana’a, Yemen

**Keywords:** Soil-transmitted helminths, Ascaris, Trichuris, Hookworm, Orang Asli, Malaysia

## Abstract

**Background:**

Despite the continuous efforts to improve the quality of life of Orang Asli (Aborigines) communities, these communities are still plagued with a wide range of health problems including parasitic infections. The first part of this study aimed at determining the prevalence of soil-transmitted helminth (STH) infections and identifying their associated factors among rural Orang Asli children.

**Methods:**

A cross-sectional study was carried out among 484 Orang Asli children aged ≤ 15 years (235 females and 249 males) belonging to 215 households from 13 villages in Lipis district, Pahang, Malaysia. Faecal samples were collected and examined by using formalin-ether sedimentation, Kato Katz and Harada Mori techniques. Demographic, socioeconomic, environmental and behavioural information were collected by using a pre-tested questionnaire.

**Results:**

Overall, 78.1% of the children were found to be infected with one or more STH species. The prevalence of trichuriasis, ascariasis and hookworm infections were 71.7%, 37.4% and 17.6%, respectively. Almost all, three quarters and one fifth of trichuriasis, ascariasis and hookworm infections, respectively, were of moderate-to-heavy intensities. Multiple logistic regression analysis showed that age of ≥ 6 years (school-age), using unsafe water supply as a source for drinking water, absence of a toilet in the house, large family size (≥ 7 members), not washing hands before eating, and not washing hands after defecation were the key factors significantly associated with STH among these children.

**Conclusion:**

This study reveals an alarmingly high prevalence of STH among Orang Asli children and clearly brings out an urgent need to implement school-based de-worming programmes and other control measures like providing a proper sanitation, as well as a treated drinking water supply and proper health education regarding good personal hygiene practices. Such an integrated control program will help significantly in reducing the prevalence and intensity of STH in Orang Asli communities.

## Background

Soil-transmitted helminth (STH) infections are still considered to be the most prevalent infections of humankind. *Ascaris lumbricoides*, hookworm (*Ancylostoma duodenale* and *Necator americanus*) and *Trichuris trichiura* are the most common STH species with global prevalence of about 1000, 900 and 500 million cases, respectively [1,2]. Nowadays, STH has been classified among the most prevalent neglected tropical diseases (NTDs) as they persist exclusively in the poorest populations often living in remote, rural areas, urban slums or in conflict zones, and have been largely eliminated elsewhere and thus are often forgotten [3].

It is estimated that STH together with schistosomiasis represent about 40% of the disease burden caused by all tropical diseases, excluding malaria [4]. However, the morbidity caused by STH is most commonly associated with infections of moderate-to-heavy intensities [5,6]. Several studies have revealed the impact of STH infections as significant predictors of protein-energy malnutrition, iron deficiency anaemia (IDA), vitamin A deficiency (VAD) and poor academic performance among schoolchildren in different countries [7-10]. Moreover, these consequences may continue into adulthood with effects on the economic productivity which trap the communities at risk of infections in a cycle of poverty, underdevelopment and disease [11]. Hence, benefits of successful STH control programmes extend well beyond eliminating STH as they improve the nutritional and health status of the children as well as contribute to higher educational attainment, labour force participation, productivity, and income among the most vulnerable populations [12-14].

Despite the efforts and interventions to control STH infections, about 70% of school-aged children at risk of STH infections are still not covered by de-worming treatment [2]. Therefore, the global efforts towards controlling STH by targeting the de-worming of 75% of school-aged children living in endemic areas by the year 2010 was not reached and therefore, STH infections remain prevalent especially in rural areas of developing countries [2].

Malaysia has witnessed great socioeconomic and infrastructural development. However, the country is still plagued with many parasitic infections especially among impoverished rural dwellers. Although there is a significant reduction in the prevalence of STH infections in the urban areas [15], the trend in the rural areas, especially among Orang Asli populations remains largely unchanged since the 1920s, with alarming high prevalence rates and prominent morbidity [5,16-20]. Thus, STH infections continue to have negative impacts on the public health particularly among Orang Asli children, and this may indicate the need for immediate and sustained action to save the lives and future of vulnerable children. Within this context, we conducted this community-based study to determine the current prevalence and the associated key factors of STH among Orang Asli children in rural Malaysia. It is hoped that findings of this study will assist public health officials to identify effective and integrated control measures to reduce the prevalence and intensity of STH in the rural communities.

## Methods

### Study design

A cross-sectional study was conducted among Orang Asli (Aborigines) population in Lipis district, Pahang. Data collection was carried out over a period of six months, from April to September 2011. The study has two parts; determining the prevalence, distribution and associated key factors of STH infections among Orang Asli children in randomly selected households, and evaluating the knowledge, attitude and practices (KAP) toward intestinal helminth infections among the heads of these households [21].

### Study area

**Figure 1:**
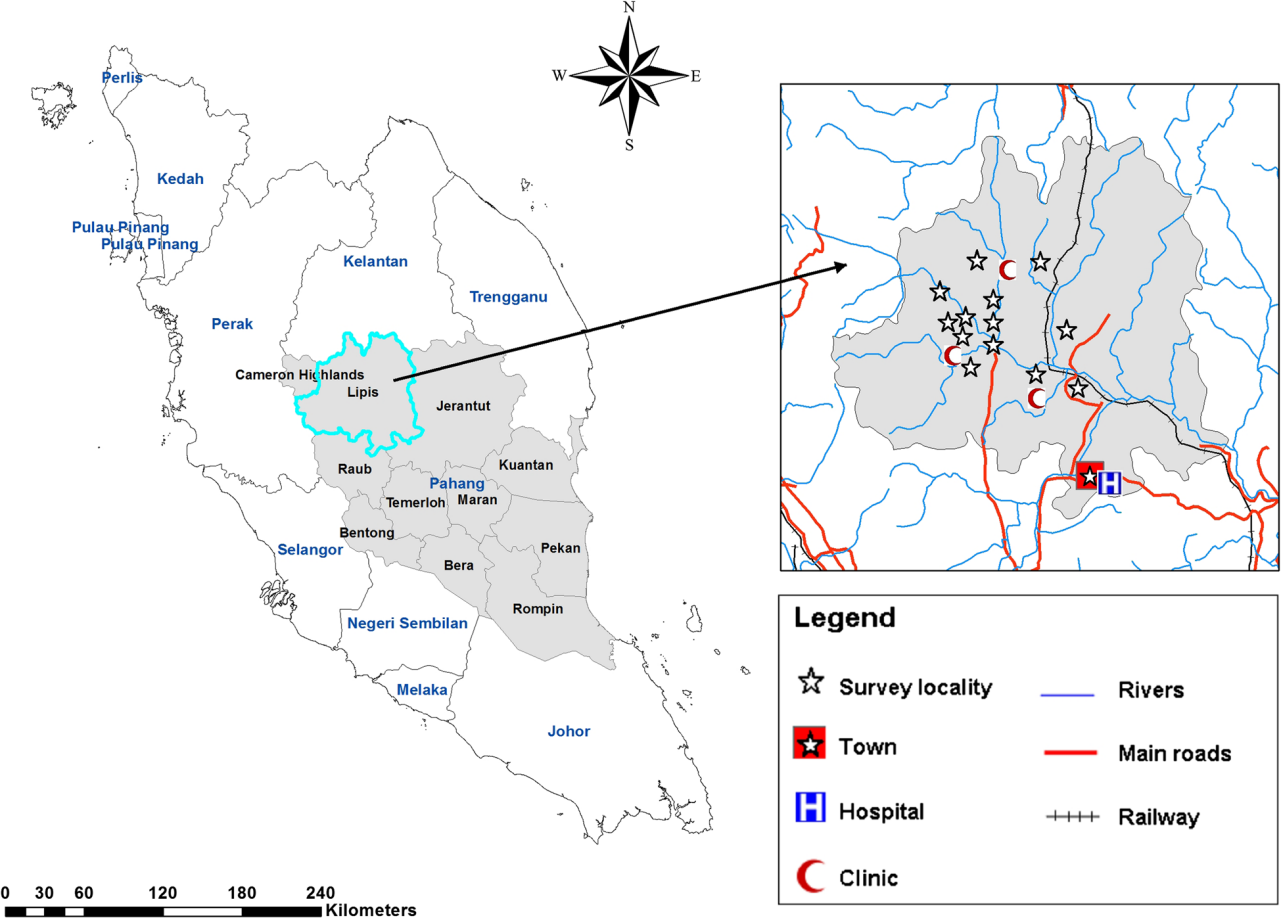
A geographic map showing Pahang state and location of the selected villages in Lipis, district.

This study was carried out in the Lipis district of Pahang state, located at the center of Peninsular Malaysia, about 200 km northeast of Kuala Lumpur with a total area of 5,198 km^2^ and a total population of 87,200 people (2010 census). The climate is equatorial with hot-humid conditions and rainfall throughout the year. The vegetation is the thick rain forest type and there are few water streams in the area. This study was conducted in 13 Orang Asli villages namely Kuala Koyan, Sentoi, Kuala Kennip, Sarang, Samut, Kuala Milut, Tual Baru, Sat Baru, Chekai (Jerankuk), Ulu Milot, Tanjung Gahai, Sungai Padi and Semoi (Figure 1). The villages were selected from the available official village list in collaboration with the Department of Orang Asli Development (JAKOA) with consideration of the following criteria: located in rural area, accessible from the main roads and each village has more than 20 houses or ≥ 100 residents.

### Study population

This study was conducted among Orang Asli people resident in the selected villages. Orang Asli are the indigenous minority peoples of Peninsular Malaysia and the name, Orang Asli, is a Malay term translated as ‘original or first people’. The total number of Orang Asli represents 0.7% of the country’s total population, and mostly they are concentrated in the different states of Malaysia, mainly Pahang, Kelantan, Perak, Selangor and Negeri Sembilan. They are generally classified under three main groups distributed all over the country; Negrito, Senoi and Proto-Malay. Each group has its own culture and language; however, most of the Orang Asli can speak Malay language which is the official national language of Malaysia. The main sub-ethnic group residing in this area is the Semai and they belong to the Senoi ethnic group.

**Figure 2:**
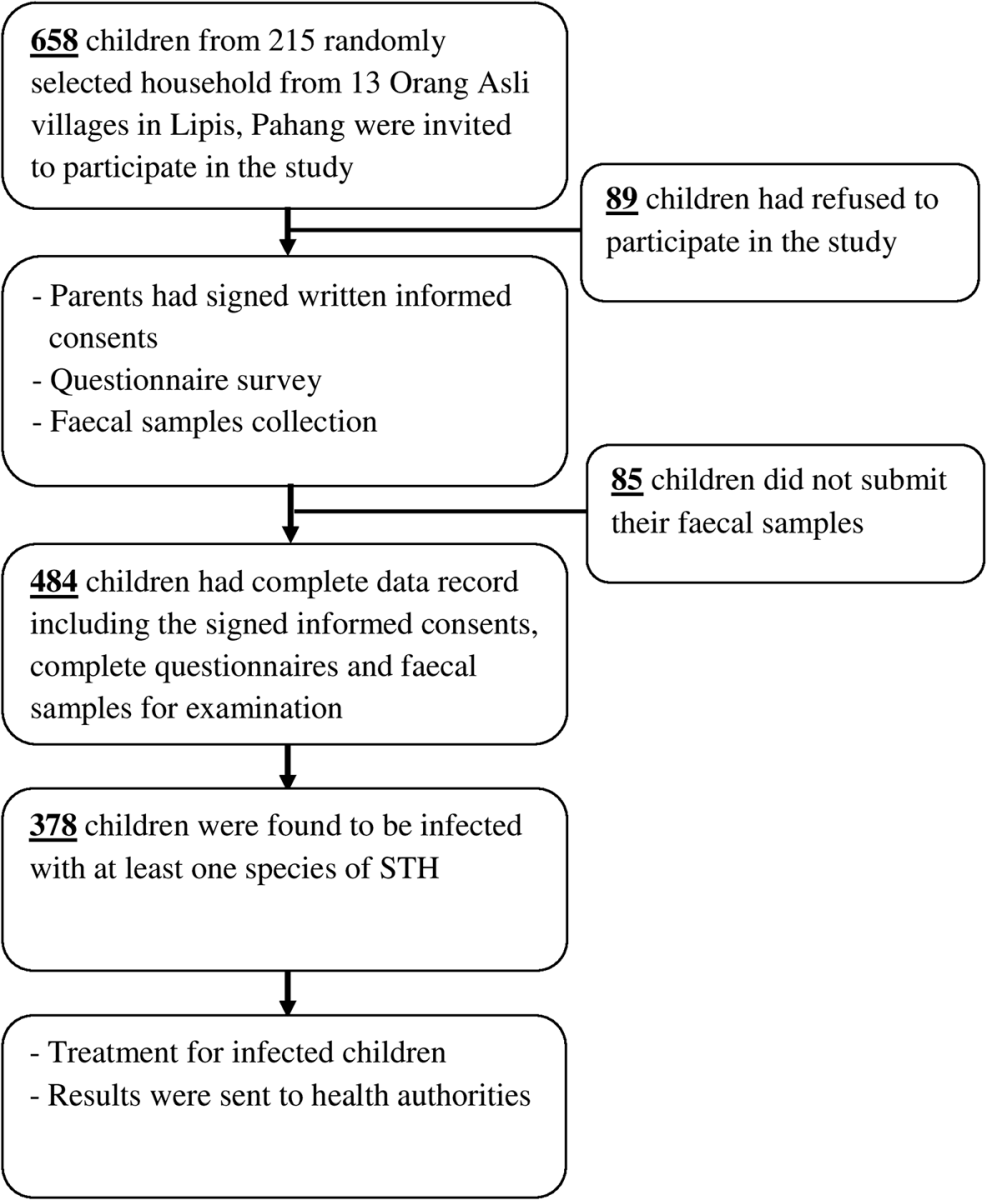
Flow chart of the participation and compliance in the present study.

Out of 710 households, 215 households were selected randomly from the villages for this study. During the visits, there were 658 children in the target age range of ≤ 15 years and all of them were invited to participate in this study. Of these children, 89 had refused to participate while 85 had not delivered stool samples in the next 2 days. Hence, 484 children had agreed voluntarily to participate in this study and delivered stool samples for examination (Figure 2).

### Questionnaire survey

A validated structured questionnaire has been designed and developed to collect information on the demographic, socioeconomic and environmental background, personal hygiene and practices, health status and the KAP of the participants towards intestinal helminth infections. Attempting to cover all possible factors associated with intestinal helminths, the questionnaire was designed in English language (original version) by the principal researchers and then translated to Malay (forward translation) by a professor of parasitology who was bilingual (English and Malay). The Malay version of the questionnaire was then back-translated to English by another bilingual professor who was also blinded to the original questionnaire. Then, the principal researchers assessed semantic equivalence between the 2 English versions (Original and the back-translated versions) for each item and made the appropriate changes of the item descriptions in the Malay version. The final Malay version was reviewed by two different experts from different institutions to check for face validity. Before starting the survey, the questionnaire was pilot tested among 28 Orang Asli people in Ulu Batu village, Selangor, Malaysia. The consistency and reliability of the questionnaire was assessed and the results showed good consistency and high test-retest reliability.

The heads of households were interviewed (face-to-face interviews) by two health assistants from JAKOA and from the Department of Parasitology, University of Malaya. Both assistants were trained on the purpose of the study and on how to administer the questionnaire. During the interviews, observation was made by another researcher on the personal hygiene of the children and household cleanliness including the availability and usage of toilets, piped water, cutting nails, wearing shoes when outside the house, washing hands and clothes.

### Parasitology

Fresh faecal samples were collected in clearly labeled containers with wide mouth and screw-caps. The children were instructed to bring their early morning stool samples the next day. The collected samples were transported for examination at the stool processing laboratory in the Department of Parasitology, University of Malaya. The examination was carried out by using three different techniques; formalin-ether sedimentation technique in order to increase the detection rates especially when the parasites are found in few numbers [22], Kato Katz technique for egg count to estimate the intensity of infections [23], and Harada Mori culture techniques to detect hookworm larvae in light infections [24]. Sample was reported as positive if eggs and/or larvae were detected by any of the three techniques. However, when the samples were negative by Kato Katz and found to be positive either by formalin-ether sedimentation technique or Harada Mori culture, the calculation of egg counts was based on the number of eggs/larvae detected by these 2 methods. To reduce human error, duplicate slides were prepared from each sample for each diagnostic technique and the slides were read by two different microscopists. The average of the readings was used in this report. The intensity of infection was recorded as eggs per gram (epg) of faeces and it was graded according to the criteria proposed by the WHO [23].

### Data analysis

Data was double-entered by two different researchers into Microsoft Office Excel 2007 spreadsheets. Then, a third researcher cross-checked the two data sets for accuracy and created a single data set. Data analysis was performed by using *Statistical Package for Social Sciences for Windows* (SPSS) version 13. Demographic, socioeconomic, environmental and behavioural characteristics were treated as categorical variables and presented as frequencies and percentages. Pearson’s Chi Square test and Fisher’s Exact test were used to test the associations of STH prevalence with demographic, socioeconomic, environmental and behavioural factors. Odd ratios (OR) and 95% confidence intervals (CI) were computed. In order to identify the risk factors significantly associated with each STH infection (*Ascaris*, *Trichuris* and hookworm), all variables that showed associations with *P* ≤ 0.25 in the univariate analysis were used to develop a multivariate logistic regression model as suggested by Bendel and Afifi [25].

### Ethical consideration

The protocol of this study was approved by the Medical Ethics Committee of the University of Malaya Medical Centre, Malaysia (Reference Number: 788.74). Before the commencement of the study, meetings were held with the head of each village to provide information about the objectives and protocol of the study and their consents were obtained. During fieldwork, the objectives and procedures of the study were explained to the heads of the sampled households. They were informed that their participation was totally voluntary and that they could decide to withdraw from the study at any time without giving any reason whatsoever. Thus, written and signed or thumb-printed informed consents were obtained from all adult participants before starting the survey. Similarly, written and signed or thumb-printed informed consents were taken from parents or guardians, on behalf of their children. All the infected children were treated with a single dose of 400 mg albendazole (Zentel®) tablets. Each child chewed the tablets before swallowing them with some water, while being observed by a researcher and medical officer (Direct Observed Therapy) [23].

## Results

### General characteristics of the households

Four hundred and eighty four children aged between 1 and 15 years, with a median age of 7 years (IQR 4–10), and living in 215 households from 13 villages in Lipis, Pahang had participated in this study. Of those, 51.4% were males and 48.6% were females. The age-group distribution showed that 60.5% were aged ≥ 6 years while 39.5% were < 6 years of age. About two thirds of the fathers had at least primary education with 16.3% of them having secondary education. On the other hand, about half of the mothers had primary education while 17.7% had secondary education. Moreover, about one third of the households had low monthly income (<RM500). Most of the houses were made of wood or bamboo and raised on stilts with palm thatched roof and bamboo planks for the wall and floor. About half of the houses had piped water supply (gravity-fed) while 89.8% had electricity. Other sources of water supply for drinking, cooking and washing included rivers, rain and deep wells. Moreover, about half of the houses had toilets; most of the toilets were pour flush toilets connected to a sewer or a septic tank, and 25% were pit latrines. The majority of Orang Asli people were engaged in agriculture, forestry, fishing and related occupations. A high proportion of them were employed as workers in rubber and oil palm plantations while about 5% were farmers. The general characteristics of the children who participated in this study are shown in Table 1.

**Table 1:** General characteristics of Orang Asli children who participated in the study (n = 484)

**Characteristics**	**n (%)**
**Age groups**	
< 6 years (pre-school)	191 (39.5)
≥ 6 years (school-age)	293 (60.5)
**Gender**	
Males	249 (51.4)
Females	235 (48.6)
**Socioeconomic status**	
Fathers’ education level (at least primary)	331 (66.6)
Mothers’ education level (at least primary)	264 (52.1)
Low household income (<RM500)	335 (65.8)
Working fathers	120 (24.1)
Working mothers	23 (4.5)
Large family size (≥ 7 members)	308 (60.5)
Piped water supply	263 (51.3)
Electricity	473 (92.7)
Presence of toilet in house	274 (53.8)

All values are number (%). RM, Malaysian Ringgit; (US$1 = RM3.15).

### Prevalence and distribution of STH infections

Faecal samples were examined for the presence of intestinal helminths including STH eggs or larvae. Overall, 378 (78%) children were found to be infected with at least one species of STH. The result as shown in Table 2, indicated that the predominant species was *T. trichiura* with a prevalence rate of 71.7%, followed by *A. lumbricoides* (37.4%) and then hookworms (17.6%). The prevalence of these 3 species increased with age. On the other hand, the prevalence of STH infections among males and females was almost similar. Moreover, 39% of STH infections were single infections with only 1 species, while the remaining 61% were mixed infections by a combination of 2 or 3 species. Ascariasis and trichuriasis were the most prevalent co-infection representing 22% of the overall prevalence followed by the combination of the triad of helminths (9.0%).

**Table 2:** Prevalence and intensity of STH infections among Orang Asli children in Lipis, Pahang (n = 484)

Intensity of infection*	**Type of infection**								
	**Trichuriasis**			**Ascariasis**			**Hookworm infection**		
	**N**	**%**	**Mean (epg)**	**n**	**%**	**Mean (epg)**	**n**	**%**	**Mean (epg)**
Light	3	0.9	900	40	22.1	3,392	69	81.2	647
Moderate	232	66.9	4,849	126	69.6	18,429	8	9.4	2,704
Heavy	112	32.3	17,441	15	8.3	60,528	8	9.4	6,405
Overall	347	71.1	8,868	181	37.4	18,594	85	17.6	1,339

* Classified according to the criteria proposed by WHO [21]. epg, Eggs per gram.

Almost one third (32.3%) and two-thirds (66.9%) of the trichuriasis were of heavy and moderate intensity, respectively (Table 2). Similarly, 8.3% and 69.6% of the infections by *A. lumbricoides* were of heavy and moderate intensity, respectively. Moreover, the majority of hookworm cases were of light intensity (81.2%). Moreover, *Giardia intestinalis* and *Entamoeba histolytica/dispar* were detected in 17.6% and 15% of the samples, respectively.

### Factors associated with STH infections

Results of univariate and multivariate analyses for the association of STH infections (trichuriasis, ascariasis and hookworm infection) with demographic, socioeconomic, environmental and behavioural factors are shown in Tables 3, 4, 5 and 6.

**Table 3:** Univariate analysis of factors associated with trichuriasis among Orang Asli children in Lipis, Pahang (n = 484)

**Variables**	**Trichuriasis**		**OR(95% CI)**	**P value**
	**No. examined**	**Infected**		
		**n (%)**		
**Age**				
School-age	293	236 (80.5)	3.0 (2.0, 4.5)	<0.001*
Pre-school-age	191	111 (58.1)	1	
**Gender**				
Male	249	176 (70.7)	0.9 (0.6, 1.3)	0.611
Female	235	171 (72.8)	1	
**Father’s educational levels**				
Non educated (< 6 years)	159	109 (68.6)	0.8 (0.5, 1.2)	0.319
Educated (≥ 6 years)	314	229 (72.9)	1	
**Mother’s educational levels**				
Non educated (< 6 years)	233	172 (73.8)	1.2 (0.8, 1.8)	0.291
Educated (≥ 6 years)	249	173 (69.5)	1	
**Father’s employment status**				
Not working	360	250 (69.4)	1.6 (0.9, 2.6)	0.083
Working	113	88 (77.9)	1	
**Mother’s employment status**				
Not working	463	333 (71.9)	0.7 (0.3, 1.7)	0.406
Working	19	12 (63.2)	1	
**Household monthly income**				
< RM500	322	234 (72.7)	1.2 (0.8, 1.8)	0.501
≥ RM500	162	113 (69.8)	1	
**Family size**				
≥ 7 members (large)	298	212 (71.1)	0.9 (0.6, 1.4)	0.732
< 7 members	186	135 (72.6)	1	
**Presence of toilet in house**				
No	218	151 (69.3)	0.8 (0.5, 1.2)	0.283
Yes	266	196 (73.7)	1	
**Presence of domestic animals**				
Yes	363	262 (72.2)	1.1 (0.7, 1.7)	0.683
No	121	85 (70.2)	1	
**Source of drinking water**				
Unsafe source (river, rain)	236	190 (80.5)	2.4 (1.6, 3.6)	<0.001*
Safe source (pipe)	248	157 (63.3)	1	
**Washing hands before eating**				
No	279	204 (73.1)	1.2 (0.8, 1.8)	0.417
Yes	205	143 (69.8)	1	
**Washing hands after defecation**				
No	191	147 (77.0)	1.6 (1.0, 2.4)	0.038*
Yes	293	200 (68.3)	1	
**Indiscriminate defecation**				
Yes	323	225 (69.7)	0.7 (0.5, 1.1)	0.159
No	161	122 (75.8)	1	
**Eating soil habit (Geophagy)**				
Yes	120	93 (77.5)	1.5 (0.9, 0.2)	0.104
No	364	254 (69.8)	1	
**Cutting nails periodically**				
No	235	166 (70.6)	0.9 (0.6, 1.3)	0.616
Yes	249	181 (72.7)	1	
**Wearing shoes when outside**				
No	191	149 (78.0)	1.7 (1.1, 2.6)	0.013*
Yes	293	198 (67.6)	1	
**Washing fruits before eating**				
No	268	191 (71.3)	1.0 (0.6, 1.4)	0.817
Yes	216	156 (72.2)	1	
**Washing vegetables before eating**				
No	156	113 (72.4)	1.1 (0.7, 1.6)	0.803
Yes	328	234 (71.3)	1	
**Boiling water before drinking**				
No	151	110 (72.8)	1.1 (0.7, 1.7)	0.704
Yes	160	120 (75.0)	1	

RM, Malaysian Ringgit; (US$1 = RM3.15). OR, Odds ratio. CI, Confidence interval. * Significant association (*P* < 0.05).

**Table 4:** Univariate analysis of factors associated with ascariasis among Orang Asli children in Lipis, Pahang (n = 484)

**Variables**	**Ascariasis**		**OR (95% CI)**	**P value**
	**No. examined**	**Infected**		
		**n (%)**		
**Age**				
School-age	293	126 (43.0)	1.9 (1.3, 2.8)	0.002*
Pre-school-age	191	55 (28.8)	1	
**Gender**				
Male	249	92 (36.9)	1.0 (0.7, 1.4)	0.834
Female	235	89 (37.9)	1	
**Father’s educational levels**				
Non educated (< 6 years)	159	60 (37.7)	1.0 (0.7, 1.5)	0.973
Educated (≥ 6 years)	314	119 (37.9)	1	
**Mother’s educational levels**				
Non educated (< 6 years)	233	88 (37.8)	1.0 (0.7, 1.5)	0.924
Educated (≥ 6 years)	249	93 (37.3)	1	
**Father’s employment status**				
Not working	360	133 (36.9)	1.2 (0.8, 1.8)	0.472
Working	113	46 (40.7)	1	
**Mother’s employment status**				
Not working	463	175 (37.8)	0.8 (0.3, 2.1)	0.583
Working	19	6 (31.6)	1	
**Household monthly income**				
< RM500	322	121 (37.6)	1.0 (0.7, 1.5)	0.908
≥ RM500	162	60 (37.0)	1	
**Family size**				
≥ 7 members (large)	298	122 (40.9)	1.5 (1.0, 2.2)	0.041*
< 7 members	186	59 (31.7)	1	
**Presence of toilet in house**				
No	218	97 (44.5)	1.7 (1.2, 2.5)	0.003*
Yes	266	84 (31.6)	1	
**Presence of domestic animals**				
Yes	363	138 (38.0)	1.1 (0.7, 1.7)	0.625
No	121	43 (35.5)	1	
**Source of drinking water**				
Unsafe source (river, rain)	236	103 (43.6)	1.7 (1.2, 2.5)	0.006*
Safe source (pipe)	248	78 (31.5)	1	
**Washing hands before eating**				
No	279	124 (44.4)	2.1 (1.4, 3.1)	<0.001*
Yes	205	57 (27.8)	1	
**Washing hands after defecation**				
No	191	97 (50.8)	2.6 (1.8, 3.8)	<0.001*
Yes	293	84 (28.7)	1	
**Indiscriminate defecation**				
Yes	323	127 (39.3)	1.3 (0.9, 1.9)	0.216
No	161	54 (33.5)	1	
**Eating soil habit (Geophagy)**				
Yes	120	47 (39.2)	1.1 (0.7, 1.7)	0.644
No	364	134 (36.8)	1	
**Cutting nails periodically**				
No	235	90 (38.3)	1.1 (0.8, 1.6)	0.691
Yes	249	91 (36.5)	1	
**Wearing shoes when outside**				
No	191	79 (41.4)	1.3 (0.9, 1.9)	0.146
Yes	293	102 (34.8)	1	
**Washing fruits before eating**				
No	268	102 (38.1)	1.1 (0.7, 1.5)	0.737
Yes	216	79 (36.6)	1	
**Washing vegetables before eating**				
No	156	67 (42.9)	1.4 (0.9, 2.1)	0.082
Yes	328	114 (34.8)	1	
**Boiling water before drinking**				
No	151	60 (39.7)	1.2 (0.8, 1.7)	0.474
Yes	333	121 (36.3)	1	

RM, Malaysian Ringgit; (US$1 = RM3.15). OR, Odds ratio. CI, Confidence interval. * Significant association (*P* < 0.05).

**Table 5:** Univariate analysis of factors associated with hookworm infection among Orang Asli children in Lipis, Pahang (n = 484)

**Variables**	**Hookworm infection**		**OR(95% CI)**	**P value**
	**No. examined**	**Infected**		
		**n (%)**		
**Age**				
School-age	293	69 (23.5)	3.4 (1.9, 6.0)	< 0.00*
Pre-school-age	191	16 (8.4)	1	
**Gender**				
Male	249	49 (19.7)	1.4 (0.8, 2.2)	0.208
Female	235	36 (15.3)	1	
**Father’s educational levels**				
Non educated (< 6 years)	159	30 (18.9)	1.2 (0.7, 1.9)	0.591
Educated (≥ 6 years)	314	53 (16.9)	1	
**Mother’s educational levels**				
Non educated (< 6 years)	233	43 (18.5)	1.2 (0.7, 1.8)	0.565
Educated (≥ 6 years)	249	41 (16.5)	1	
**Father’s employment status**				
Not working	360	63 (17.5)	1.0 (0.6, 1.8)	0.961
Working	113	20 (17.7)	1	
**Mother’s employment status**				
Not working	463	83 (17.9)	0.3 (0.1, 1.9)	0.154
Working	19	1 (5.3)	1	
**Household monthly income**				
< RM500	322	54 (16.8)	0.9 (0.5, 1.4)	0.519
≥ RM500	162	31 (19.1)	1	
**Family size**				
≥ 7 members (large)	298	53 (17.8)	1.0 (0.6, 1.7)	0.870
< 7 members	186	32 (17.2)	1	
**Presence of toilet in house**				
No	218	51 (23.4)	2.1 (1.3, 3.4)	0.002*
Yes	266	34 (12.8)	1	
**Presence of domestic animals**				
Yes	363	67 (18.5)	1.3 (0.7, 2.3)	0.370
No	121	18 (14.9)	1	
**Source of drinking water**				
Unsafe source (river, rain)	236	49 (20.8)	1.5 (0.9, 2.5)	0.071
Safe source (pipe)	248	36 (14.5)	1	
**Washing hands before eating**				
No	279	61 (21.9)	2.1 (1.3, 3.5)	0.004*
Yes	205	24 (11.7)	1	
**Washing hands after defecation**				
No	191	32 (16.8)	0.9 (0.6, 1.5)	0.706
Yes	293	53 (18.1)	1	
**Indiscriminate defecation**				
Yes	323	67 (20.7)	2.1 (1.2, 3.6)	0.009*
No	161	18 (11.2)	1	
**Eating soil habit (Geophagy)**				
Yes	120	25 (20.8)	1.3 (0.8, 2.2)	0.277
No	364	60 (16.5)	1	
**Cutting nails periodically**				
No	235	37 (15.7)	0.8 (0.5, 1.3)	0.307
Yes	249	48 (19.3)	1	
**Wearing shoes when outside**				
No	191	38 (19.9)	1.3 (0.8, 2.1)	0.276
Yes	293	47 (16.0)	1	
**Washing fruits before eating**				
No	268	46 (17.2)	0.9 (0.6, 1.5)	0.798
Yes	216	39 (18.1)	1	
**Washing vegetables before eating**				
No	156	21 (13.5)	0.6 (0.4, 1.1)	0.102
Yes	328	64 (19.5)	1	
**Boiling water before drinking**				
No	151	34 (22.5)	1.6 (0.99, 2.6)	0.054
Yes	333	51 (15.3)	1	

RM, Malaysian Ringgit; (US$1 = RM3.15). OR, Odds ratio. CI, Confidence interval. * Significant association (*P* < 0.05).

**Table 6:** Multivariate analysis of factors associated with STH infections among Orang Asli children in Lipis, Pahang (n = 484)

**Variables**	**Trichuriasis**			**Ascariasis**			**Hookworm infections**		
	**Adjusted OR**	**95% CI**	***P***	**Adjusted OR**	**95% CI**	***P***	**Adjusted OR**	**95% CI**	***P***
School-age	3.6	2.3, 5.5	<0.001	1.9	1.3, 2.9	0.003	3.6	1.9, 6.5	<0.001
Absence of toilet in house	-	-	-	1.8	1.2, 2.6	0.005	2.1	1.3, 3.5	0.003
Source of drinking water (unsafe water)	2.9	1.9, 4.5	<0.001	2.2	1.4, 3.2	<0.000	1.7	1.1, 2.9	0.032
Not washing hands before eating	-	-	-	-	-	-	2.2	1.3, 3.8	0.004
Not washing hands after defecation	-	-	-	2.6	1.8, 3.9	<0.001	-	-	-
Large family size (≥7 members)	-	-	-	1.7	1.1, 2.5	0.016	-	-	-

OR, Odds ratio. CI, Confidence interval.

Table 3 shows that the prevalence of trichuriasis was significantly higher among school-age children (80.5%; 95% CI = 75.6, 84.7) when compared with pre-school children (58.1%; 95% CI = 51.0, 64.9). Similarly, children who use unsafe sources for drinking water had higher prevalence (80.5%; 95% CI = 75.0, 85.1) when compared to those who use piped water (63.3%; 95% CI = 57.2, 69.1). Moreover, the prevalence of infection was significantly higher among children who do not wash their hands after defecation (77.0%; 95% CI = 73.3, 82.4) and those who walk barefooted (78.0%; 95% CI = 72.7, 83.3) when compared to those who practise hand washing after defecation (68.3%; 95% CI = 62.7, 72.6) and those always wear shoes when outside the house (67.6%; 95% CI = 62.0, 71.6). The results of multiple logistic regression for the significant factors (Table 6) confirmed that school-age children were at higher odds for *Trichuris* infection when compared with pre-school children by 3.6 times (OR = 3.6; 95% CI = 2.3, 5.5). Moreover, children who used unsafe sources for drinking water had significantly higher odds of having trichuriasis when compared to those living in houses supplied with piped water (OR = 2.9; 95% CI = 1.9, 4.5).

Table 4 shows that the prevalence of ascariasis among school-age children (43.0%; 95% CI = 37.5, 48.7) was significantly higher than the prevalence among pre-school children (28.8%; 95% CI = 22.9, 35.6). Similarly, the prevalence of ascariasis among children who live in families with ≥ 7 members (40.9%; 95% CI = 37.7, 46.6) was significantly higher than the prevalence among those living in smaller families with < 7 members (31.7%; 95% CI = 25.5, 36.2). Moreover, the prevalence of ascariasis was significantly higher among children who live in houses without toilets (44.5%; 95% CI = 38.1, 51.1) and those using unsafe sources of drinking water (43.6%; 95% CI = 38.1, 50.0) when compared to those living in houses with toilets (31.6%; 95% CI = 26.3, 37.4) and piped water supply (31.5%; 95% CI = 26.0, 37.2). A significant association between ascariasis and personal hygiene practices was also reported as the prevalence was found to be higher among children who do not wash their hands before eating (44.4%; 95% CI = 38.7, 50.3) when compared to those who practise hand washing before eating (27.8%; 95% CI = 22.1, 34.3). Similarly, children who do not wash their hands after defecation (50.8%; 95% CI = 43.8, 57.8) had significantly higher prevalence of ascariasis when compared with those who wash their hands (28.7%; 95% CI = 23.8, 34.1).

Five factors associated significantly with ascariasis were retained by multiple logistic regression model analysis (Table 6). School-age children were at twice the odds of *Ascaris* infection when compared with pre-school children (95% CI = 1.3, 2.9). Similarly, not washing hands after defecation increased children’s odds for ascariasis when compared with always washing hands after defecation by 2.6 times (95% CI = 1.8, 3.9). Children who did not have piped water facility in their houses had 2.2 (95% CI = 1.4, 3.2) times the odds of having *Ascaris* infection. Likewise, children who lived in houses without functioning toilets were found to have higher odds of having *Ascaris* infection as compared with their counterparts (OR = 1.7; 95% CI = 1.2, 2.6). Moreover, children of families with ≥ 7 members had significantly higher odds (OR = 1.7; 95% CI = 1.1, 2.5) of having *Ascaris* infection as compared to those who live in families with < 7 members.

Table 5 shows that school-age children had significantly higher prevalence of hookworm infection (23.5%; 95% CI = 19.1, 28.7) when compared to pre-school children (8.4%; 95% CI = 5.2, 13.2). Similarly, the prevalence of hookworm among children who lived in houses without toilets (23.4%; 95% CI = 18.3, 29.4) and those who did not practise hand washing before eating (21.9%; 95% CI = 17.4, 27.1) was almost twice as much as those having toilets in their houses (12.8%; 95% CI = 9.3, 17.3) and those that wash their hands before eating (11.7%; 95% CI = 8.0, 16.8). Moreover, the results showed that the prevalence of infection was significantly associated with indiscriminate defecation, the prevalence of hookworm infection was significantly higher among children who used nearby rivers or bushes for defecation (20.7%; 95% CI = 17.2, 25.7) when compared to those who use the toilets (11.2; 95% CI = 7.0, 16.4). Although the prevalence of hookworm was higher among those who do not boiling drinking water ( 22.5%) compared to those who practise boiling it (15.3%) and among children living in houses without safe sources for drinking water (20.8%) when compared to those who live in houses with piped water supply (11.7%), these differences were not statistically significant (*P* > 0.05).

Multiple logistic regression model retained 4 factors associated significantly with hookworm infection (Table 6). Those of school-age were at greater odds for hookworm infection by 3.6 times as compared to pre-school children (95% CI = 1.9, 6.5). Similarly, the results showed that children who live in houses without functioning toilets had 2.1 times the odds (OR = 2.1; 95% CI = 1.3, 3.5), using unsafe sources for drinking water had 1.7 times odds (OR = 1.7; 95% CI = 1.1, 2.9), and not washing hands before eating had 2.2 times odds (OR = 2.2; 95% CI = 1.3, 3.8) of having hookworm infection when compared with their counterparts.

## Discussion

Soil-transmitted helminth (STH) infection remains a major public health problem among the Malaysian Orang Asli population, thereby possibly contributing to their overall backwardness and low productivity. The adherence of these people to be confined within the rainforest away from other people possibly contributes to their limited integration into Malay society, poor public enlightenment and low level of education. The findings of the present study showed high prevalence of STH infections with 78% of the children was infected with at least one STH species. The predominant parasite was *T. trichiura,* followed by *A. lumbricoides,* while hookworm was the least prevalent. Although, *Strongyloides stercoralis* larvae were detected in 7.1% of soil samples in the study area (Lipis district), *S. stercoralis* infection is not endemic in Malaysia and it usually occurs as sporadic cases [26,27]. These findings are consistent with the reports from previous studies among Orang Asli people in Malaysia [5,16,17,28]. However, our findings were contrary to some recent reports from neighbouring countries such as Thailand (mainly southern region) where hookworms were reported as the most prevalent species [29,30] and China where ascariasis was most prevalent [31].

The high prevalence rates of *T. trichiura* infection reported in this study could be attributed to the low efficacy of the benzimidazole anthelminthics drugs against this worm as reported previously [32,33]. The adult worm usually live embedded in the walls of the lumen of their host, hence it is difficult to be killed and expelled by a single dose of anthelminthics. Researchers therefore fear that anthelminthics resistance might be emerging in *T. trichiura*[34,35]. On the other hand, the low prevalence rate of hookworm reported in our study might be connected to the nature of the soil in several parts of Malaysia which is the heavy clayey-loam type. This type of soil was found to be unsuitable for hookworm larval development [36,37].

The findings of the current study also revealed that almost all, three quarters and one fifth of the total infections by *T. trichiura*, *A. lumbricoides* and hookworm, respectively, were of moderate-to-heavy intensities. This is higher than the prevalence reported by previous studies conducted among the Orang Asli [5,16,17]. This prevalence is alarming especially considering the fact that clinical manifestations and other consequences of these infections tend to be positively correlated with the burden of infection [5,6,31]. Besides the continuous exposure to the infections in these communities, the majority of the children did not receive any anthelmintic drugs in the last 12 months. In the absence of effective control and preventive measures, it is more likely that STH infections will continue to have devastating consequences and public health implications in these communities.

The World Health Organization is aware that the elimination of STH infections in endemic communities around the world may not be a feasible proposition; hence effort is geared towards the reduction of prevalence and intensity of infection to a low level. Endemic communities for STH are classified into 3 transmission categories for the adoption of treatment strategy in preventive chemotherapy; category I (high), category II (medium), and category III (low) [2]. Based on this classification, our study area and most probably all Orang Asli areas in Peninsular Malaysia fall within the first category (high risk communities), with STH prevalence of more than 50% and more than 10% of the infections being of heavy intensity. Hence, this high prevalence calls for urgent interventions particularly considering the fact that these infections lead to malnutrition and growth retardation [7,31], poor school performance [38], high school absenteeism rate [39], IDA and VAD [40,41], and overall poor productivity [42].

The present study investigated the possible factors associated with STH infections (trichuriasis, ascariasis and hookworm infection) among the studied children and revealed that age (school-age), absence of toilet and piped water supply in the household, large family size (≥ 7 members), and not washing hands before eating and after defecation are the key factors that found to be associated with the three STH species. Many previous studies, including our previous published reports, have investigated and presented the risk factors of STH in different ways; either for overall intestinal parasitic infections (protozoa & helminth) or for only one species of STH or for the heavy burden of overall STH infections. In order to develop an integrated control programme, the current study attempted to provide a complete picture and identified the significant associated key factors of the three STH species within the same community. However, our findings are in agreement with previous studies conducted in Malaysia [5,16,28] and abroad [30,43-46]. School-age children may have more exposure to the sources of infections due to their excessive mobility as compared to the pre-school children who usually receive more parental care. Moreover, if school environment is unhygienic this may also contribute to the transmission of these parasites.

In addition, poor personal hygiene including not washing hands before eating and after defecation is well documented as a significant risk factor of intestinal parasitic/bacterial/viral infections [45,47]. Given the fact that the infective stages of these helminths are found in soil, washing hands before eating will help significantly in preventing these infections especially among children who love to play with soil.

In Malaysia, all Orang Asli communities are located close to rivers which are considered essential for Orang Asli life as they use water from streams for most of their daily activities (swimming, cooking, drinking, bathing and washing). However, rivers are also their preferred site for defecation and the practice of defecating near the streams by the residents (especially children) in these communities has been noted by other workers [5,28]. Thus, the untreated water is always likely to be contaminated with parasites eggs and/or cysts and its usage for household activities enhances the likelihood of infections. Moreover, the lack of functioning toilet facilities in the house contributes to the spread of intestinal parasitic infections. Overall, our findings showed that school-age, using unsafe sources for drinking water and lack of toilets in the house increases the odds of *Trichuris*, *Ascaris* and hookworm infections by about 2 to 3 times among these children.

Our findings also showed that children who belong to large families (≥ 7 members) were at higher odds of *Ascaris* infection compared to children from smaller families. This finding is consistent with previous studies [16,48]. The horizontal spread or the focal transmission of infection among family members in the vicinity of the home may explain this finding. Moreover, a significant association between intestinal parasitic infections and the presence of other infected family members was reported [49].

The associated key factors identified by the present study are the main focus of effective STH control programmes [4]. In Malaysia, the national mass de-worming programme using a single dose of pyrantel pamoate once or twice a year was discontinued in 1983 due to the low effectiveness of the drug against *Trichuris* and hookworm. However, children in some rural areas are still receiving albendazole tablets. This is an intermittent distribution, without any monitoring system, of anthelmintics by researchers and community health campaigns by the Ministry of Health. This practice is not recommended as it may contribute to the emergence of anthelmintics drug resistance. Moreover, the re-infection rates of STH were reported to be high and by 6 months after complete de-worming the prevalence and intensity of infections were similar to pre-treatment levels [50,51].

Orang Asli communities in Peninsular Malaysia share similar socioeconomic, environmental and health profiles. Our study provides a community-based picture of STH status among children with a poor socioeconomic, environmental and personal hygiene background. Thus, we may speculate that the findings of the present study can be generalised to rural Orang Asli children in other states. On the other hand, these results may not be generalisable to the entire Malaysian rural population as ethnic groups other than Orang Asli have a better socioeconomic and environmental situation. However, further investigations are required to confirm these conjectures.

## Conclusions

This study reveals an alarmingly high prevalence of STH among Orang Asli children and this supports an urgent need to start an integrated and effective STH control programme. School-age, lack of toilets and piped water supply in the house, large family size (≥ 7 members), and not washing hands before eating and after defecation were the key factors significantly associated with STH infections in the studied population. Based on these findings, implementing periodic school-based de-worming programmes, providing proper sanitation and portable safe water supply, and providing proper health education pertinent to good personal hygiene and good sanitary practices will help in reducing the prevalence and intensity of STH in these communities. In connection with the importance of community participation in the prevention and control activities, it is essential to evaluate knowledge, attitude and practices of the concerned population before attempting to introduce any change or innovation.

## Competing interests

The authors have declared that no competing interests exist.

## Authors’ contributions

NAN was involved in all phases of the study, including data collection, data analysis, interpretation, and write-up of the manuscript; AB and HMA designed and supervised the study, and revised the analysis and manuscript. AA and MAR were involved in the collection and laboratory examination of samples. All authors read and approved the final manuscript.
